# Raising Awareness of Canine, Feline and Human Dirofilariosis in Aveiro, Portugal: A One Health Perspective

**DOI:** 10.3390/ani15070952

**Published:** 2025-03-26

**Authors:** Joana Esteves-Guimarães, José Alberto Montoya-Alonso, Jorge Isidoro Matos, Elmano Ramalheira, Elena Carretón, Ivan Rodríguez-Escolar, Alfonso Balmori-de la Puente, Manuel Collado-Cuadrado, Rodrigo Morchón, Ana Patrícia Fontes-Sousa

**Affiliations:** 1 Clínica Veterinária Aanifeira, 4520-409 Santa Maria da Feira, Portugal; joana.eg@gmail.com; 2 Internal Medicine, Faculty of Veterinary Medicine, Research Institute of Biomedical and Health Sciences (IUIBS), University of Las Palmas de Gran Canaria, 35413 Las Palmas de Gran Canaria, Spain; alberto.montoya@ulpgc.es (J.A.M.-A.); jorge.matos@ulpgc.es (J.I.M.); elena.carreton@ulpgc.es (E.C.); rmorgar@usal.es (R.M.); 3 Zoonotic Infections and One Health Group, Biomedical Research Institute of Salamanca (IBSAL), Centre for Environmental Studies and Rural Dynamization (CEADIR), Faculty of Pharmacy, University of Salamanca, Campus Miguel Unamuno, 37007 Salamanca, Spain; ivanrodriguez@usal.es (I.R.-E.); a.balmori@usal.es (A.B.-d.l.P.); manuelcollado@usal.es (M.C.-C.); 4 Clinical Pathology Department, Baixo Vouga-Public Business Entity (EPE) Hospital Centre, Avenida Artur Ravara, 3810-501 Aveiro, Portugal; elmano.ramalheira@ua.pt; 5 Centre for Pharmacological Research and Drug Innovation (MedInUP/RISE-Health), Department of Immuno-Physiology and Pharmacology, Veterinary Hospital of the University of Porto (UPVET), School of Medicine and Biomedical Sciences (ICBAS), University of Porto, Rua Jorge de Viterbo Ferreira 228, 4050-313 Porto, Portugal

**Keywords:** *Dirofilaria immitis*, *Wolbachia pipientis*, zoonosis, Aveiro, Portugal, seroepidemiological study

## Abstract

Climatic and various socio-geographical variables have significantly influenced the global spread of *Dirofilaria immitis*. The coastal district of Aveiro, Portugal, marked by its unique hydrographic structure, Ria de Aveiro, and a concerning rise in heartworm disease, was the focus of our study. We aimed to update the prevalence of *D. immitis* in dogs and the seroprevalence in cats and humans, correlating these data with epidemiological information. A total of 430 dogs were sampled for *D. immitis* antigens, and 426 cats and 398 humans for *D. immitis* and *Wolbachia* sp. antibodies. In addition, we developed and validated an infection risk map for *D.* immitis with the geolocation of positive samples. Our results indicate a canine prevalence of 4.7%, peaking at 16.7% in Vagos. Feline and human seroprevalences were 8.9% (26.7% in Espinho) and 3.0% (Vagos presented the most alarming results), respectively. Positive samples were found in both high- and low-risk areas highlighting the need for chemoprophylaxis in all municipalities. Risk factors identified included lack of vaccination and internal deworming in cats, while dogs faced risks from inadequate vaccination and outdoor exposure. Our study identifies Aveiro as an endemic area, with a need for control measures to address this public health threat.

## Simple Summary

*Dirofilaria immitis* is a parasitic nematode that causes animal and human dirofilariosis, a disease transmitted through mosquito bites. Due to socio-geographic and environmental factors, this disease is spreading globally. In Portugal, the district of Aveiro was studied following a significant recent increase in infection rates among the canine population, which serves as a reservoir. Our study assessed the prevalence of *D. immitis* infection in dogs, as well as the presence of antibodies in cats and humans, aiming to establish correlations with epidemiological variables. Positive samples were identified in all three species across almost the entire district of Aveiro, with an overall canine prevalence of 4.7% and feline and human seroprevalence rates of 8.9% and 3.0%, respectively. The lack of vaccination and internal dewormers were identified as risk factors in cats, while in dogs, the main risk factors were lack of vaccination and outdoor exposure. Our findings indicate that not only dogs but also cats and humans are being exposed to *D. immitis*, with certain municipalities, such as Vagos and Águeda, exhibiting alarming levels of parasite presence. Urgent control measures are required to mitigate the spread of this disease, which is no longer confined to veterinary species.

## 1. Introduction

*Dirofilaria immitis* is a nematode capable of causing cardiopulmonary infection in dogs and cats, completing its life cycle in mosquitoes, primarily of the *Culex*, *Aedes* and *Anopheles* genera. Following larval inoculation by a mosquito, the parasite matures into adult worms, migrating to the pulmonary vascular tree and, in severe cases, reaching the main pulmonary arteries and right side of the heart [1]. In both wild and domestic canids, this process is often prolonged, with an extended asymptomatic phase during which they serve as reservoirs. Chronic endothelial damage caused by adult worms and their bacterial endosymbiont, *Wolbachia pipientis*, contributes to pulmonary hypertension, potentially leading to right heart failure. In cases of high parasite burden, the obstruction of blood flow at the level of the right atrium and tricuspid valve induces hemolysis, and, in severe instances, results in fatal caudal vena cava syndrome [2].

*Dirofilaria immitis* can also maturate in feline hosts; however, it is believed that the feline immune system inhibits its full development [3]. Consequently, cats typically harbor no more than six adult worms, and their microfilaremia remains low and transient [4]. Clinical manifestations in cats range from asymptomatic cases to sudden death [4]. Nevertheless, most affected cats exhibit respiratory symptoms, including cough, tachypnea and respiratory distress [5], resulting from the pulmonary inflammatory response triggered by parasite migration and development [6]. The severity and presentation of these clinical signs are unpredictable [7]. In cases of aberrant migration, neurological signs may also occur [8].

Humans are accidental hosts, and *D. immitis* larvae were traditionally believed to be incapable of maturing into adult worms, as they are presumably eliminated by the host’s immune system [9]. Interestingly, however, cases of adult *D. immitis* have been reported in the parietal pleura [10], heart and inferior vena cava [11] and peritoneal cavity [12] of human patients. In most cases, human dirofilariosis caused by *D. immitis* manifests as pulmonary nodules that mimic malignancy [13,14], though larvae have also been identified in the subcutaneous tissue [15,16], eye [17,18,19], testicle [20] and liver [21]. Due to its nonspecific clinical presentation, the disease is often overlooked.

Climate change, global warming, geographical characteristics of certain regions, such as the presence of marshland and water bodies, and various socio-cultural factors create favorable conditions for vector reproduction and the spread of this disease. Beyond native mosquito species, recent climate trends have also facilitated the establishment of invasive species, such as *Ae. aegypti* and *Ae. albopictus*, both competent vectors of *D. immitis* [22]. In Portugal, *Ae. aegypti* has not yet been reported, but the evidence suggests that *Ae. albopictus* is an established mosquito species in Algarve and Penafiel [23,24], the latter being geographically close to the district of Aveiro.

Over the past decade, the prevalence of canine dirofilariosis has been rising [25,26,27,28], with new cases in previously disease-free regions, such as Cyprus [29], Cape Verde [30] and Cuba [31]. Similarly, recent studies have confirmed, for the first time, the presence of *D. immitis* in cats [32,33], reflecting a growing awareness of the disease beyond canids. With the rising prevalence of dirofilariosis, both domestic and wild canids play a key role in its transmission due to their prolonged asymptomatic phase and function as reservoirs of *Dirofilaria*, increasing the risk of human infection. Consequently, dirofilariosis persists as a significant global public health concern.

The presence of *D. immitis* in specific regions of Portugal has been studied in canine [34,35,36], feline [37,38,39] and human populations [40]. However, the data on human exposure in Aveiro district remain limited [40], despite central Portugal being an area of significant canine prevalence [41] and feline seroprevalence [37]. Moreover, a recent study on dogs identified the Aveiro district as particularly concerning, with a rising prevalence of dirofilariosis exceeding the national average [28]. This study aimed to update the prevalence of canine dirofilariosis in the Aveiro district, as well as its seroprevalence in cats and humans. Additionally, we sought to investigate potential risk factors, analyze correlations with key epidemiological variables and assess the potential risk to human health.

## 2. Materials and Methods

### 2.1. Location and Climatology

**Figure 1:**
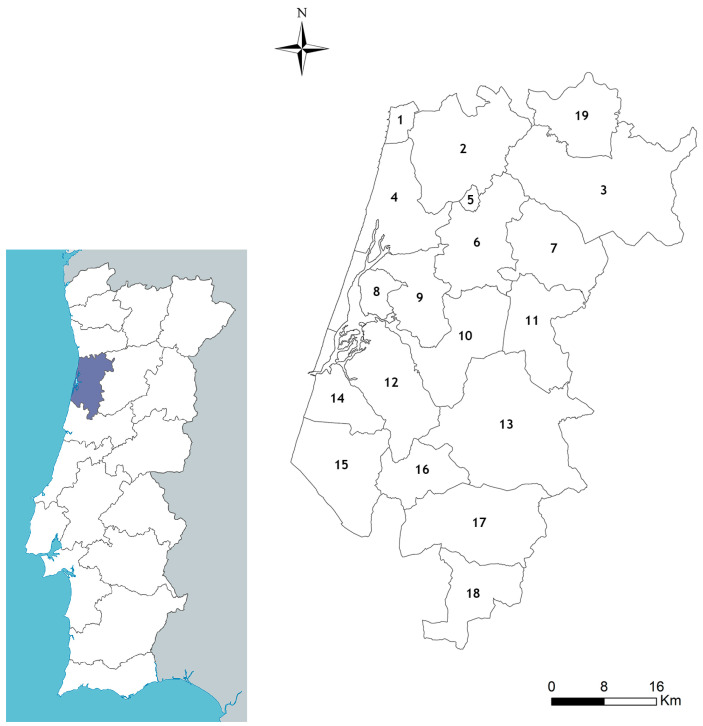
Municipalities of Aveiro in Portugal along with its main hydrographic features. The numbers correspond to the municipalities listed in Table 1.

Continental Portugal is located in the Western Iberian Peninsula, in the southern region of Western Europe. The district of Aveiro, covering approximately 2.800 km^2^, lies in the central-north coastal region of the country, featuring a 60 km long shoreline. One of its most distinctive features is the *Ria de Aveiro*, a 75 km^2^ coastal lagoon formed by an ocean retreat. This complex ecosystem, characterized by coastal ridges, lagoons and marshland, serves as a habitat for diverse species. The Aveiro district comprises the municipalities of Águeda, Albergaria-a-Velha, Anadia, Arouca, Aveiro, Castelo de Paiva, Espinho, Estarreja, Ílhavo, Mealhada, Murtosa, Oliveira de Azeméis, Oliveira do Bairro, Ovar, Santa Maria da Feira, São João da Madeira, Sever do Vouga, Vagos and Vale de Cambra (Figure 1). The *Ria de Aveiro* extends through Aveiro, Estarreja, Ílhavo, Murtosa, Ovar and Vagos. Additionally, it serves as the estuary of the Vouga River, which flows from Sever do Vouga and Águeda to Aveiro, where it branches into multiple channels across a low-lying marshy terrain.

According to the Köppen classification [42], the climate in Aveiro is temperate with a dry or temperate summer, with temperatures ranging from 0 to 18 °C during the coldest months (Csb climate). Over the past decades, the region’s median temperature has steadily increased, rising from 13 °C in the 1970s to over 14 °C in 2000s [43].

### 2.2. Samples and Assays

#### 2.2.1. Cats and Humans

This study included 426 cats from Aveiro, with samples collected between September 2020 and September 2024 from 17 participating veterinary centers. Most samples were obtained from cats undergoing routine check-ups or neutering procedures but some of them were also recruited from consultations (with clinical signs). Inclusion criteria required cats to be over 8 months old, with no history of heartworm infection or prophylactic treatments. A comprehensive record was maintained for each animal, documenting age, sex, breed, habitat and clinical history, including, when possible, the Feline Leukemia Virus (FeLV) and Immunodeficiency Virus (FIV) status, along with demographic data. For cats obtained through catch–neuter–return programs, age was estimated, and previous medical history was often incomplete or unavailable. Owners were informed about the study’s objectives and provided consent for participation. Blood samples were collected from the jugular vein for routine diagnostics and subsequently made available for this study. The samples were placed in serum tubes and centrifuged and stored −20 °C until analysis.

For this study, 398 human serum samples were collected from Centro Hospitalar do Baixo Vouga (Aveiro, Portugal) between November 2021 and May 2022. Participants were included based on their residence within the study’s target area (Aveiro district) and their informed consent to participate. Blood samples were initially collected for prescribed diagnostic purposes and subsequently made available for this research. Samples were placed in serum tubes, centrifuged and stored at −20 °C until testing. This study complies with the Declaration of Helsinki and received approval from the hospital’s ethics committee. Patient confidentiality was strictly maintained and all participants provided written informed consent.

To estimate the *D. immitis* seroprevalence in cats and humans, the serum samples were tested using in-house ELISA techniques for anti-*D. immitis* and anti-*Wolbachia* antibody detection, as described by Montoya-Alonso et al. [44] and Zumaquero et al. [45], respectively, with some modifications. In brief, the plates were coated with 0.8 μg of *D. immitis* somatic antigen and 0.3 μg of recombinant *Wolbachia* surface protein (rWSP). Serum samples were prepared at 1/100 for anti-*D. immitis* and 1/40 for anti-rWSP. Anti-human and anti-cat IgG antibody horseradish peroxidase were applied at 1/4000 dilution. The optical densities were measured in an Easy-Reader (Bio-Rad Laboratories, Hercules, CA, USA) at 492 nm. Cut-off points of ELISA *D. immitis* 0.8 and ELISA rWSP 0.5 were obtained as arithmetic mean optical density ±3 standard deviations of serum of clinically healthy cats and humans. The humans and cats were considered seropositive when anti-*D. immitis* and anti-rWSP antibodies presented jointly.

#### 2.2.2. Dogs

The study analyzed 430 blood samples from domestic dogs, both privately owned and shelter-housed, collected from 22 participating veterinary centers between September 2019 and September 2024. Clinic participation was voluntary, and sample collection occurred throughout the study period. Dogs were included based on specific criteria: they had to be over 8 months old, with no history of heartworm infection or regular chemoprophylaxis. A complete record was kept for each animal, including identification (age, sex, weight, fur length, breed and habitat), clinical history and demographic data.

Blood samples were drawn from the jugular vein and immediately processed for testing using whole blood, serum or plasma. All samples were screened for circulating *D. immitis* antigens with a commercial immunochromatographic test kit (Uranotest Dirofilaria^®^, UranoVet SL, Barcelona, Spain), following the manufacturer’s instructions.

### 2.3. Dirofilaria immitis Risk Map and Its Validation

To obtain an infection risk map for *D. immitis* in Aveiro, we used the methodology previously described by Rodríguez-Escolar et al. [46]. Indeed, we created a final habitat suitability model for *Cx. pipiens* in Aveiro with 7 bioclimatic variables and 6 environmental variables with the KUENM package in R (1.1.10). We then produced a generation number map for *D. immitis* in R-4.3.0 software. Finally, we multiplied the final habitat suitability model for *Cx. pipiens* and the number-of-generations map for *D. immitis* using the ArcMap 10.8 raster calculator (ESRI, 2020, Redlands, CA, USA). For the validation of the infection risk map for *D. immitis*, seropositive dogs, cats and humans in the Aveiro district were georeferenced and overlaid on the risk map to see which area they inhabited.

### 2.4. Statistical Analysis

The data were analyzed using R software, version 4.3.0. (R Core Team, Vienna, Austria, 2023). Descriptive analysis of the qualitative variables was carried out considering the number of cases and percentages. Pearson’s χ2 test and Fisher’s exact test (recommended for datasets with small expected frequencies for each combination cell) were performed between two categorical variables (factor and disease). The Bonferroni correction was used for multiple comparisons together with the pairwise Fisher test function in R. The odds ratio function in R was used to understand the association level between variables (higher or lower than 1). The significance level was established at *p* < 0.05.

## 3. Results

Table 1 presents the overall results for *D. immitis*, including its prevalence in dogs and seroprevalence in cats and humans, along with its distribution across the municipalities of Aveiro. Notably, no human, canine or feline samples were collected from the municipality of Castelo de Paiva. Arouca lacks both feline and human samples, while Espinho, São João da Madeira, Vale de Cambra and Mealhada have no human samples available.

**Table 1:** Seroprevalence (human, cats) and prevalence (dogs) of *Dirofilaria immitis* in human, feline and canine specimens from the different municipalities of the Aveiro district in Portugal. Abbreviations: n = number of samples; + = number of positive samples; % = percentage of positive samples; - = no samples.

	Humans			Cats			Dogs		
	n	+	%	n	+	%	n	+	%
Overall	398	12	3.0	426	38	8.9	430	20	4.7
1. Espinho	0	-	-	15	4	26.7	14	0	0
2. Santa Maria da Feira	2	0	0	84	4	4.8	31	2	6.5
3. Arouca	0	-	-	0	-	-	7	0	0
4. Ovar	1	0	0	88	13	14.8	153	9	5.9
5. São João da Madeira	0	-	-	14	0	0	17	0	0
6. Oliveira de Azeméis	1	0	0	27	2	7.4	10	0	0
7. Vale de Cambra	0	-	-	2	0	0	7	0	0
8. Murtosa	8	0	0	15	0	0	17	1	5.9
9. Estarreja	15	0	0	18	3	16.7	40	2	5.0
10. Albergaria-a-Velha	40	0	0	15	0	0	17	0	0
11. Sever do Vouga	14	1	7.1	14	2	14.3	10	0	0
12. Aveiro	154	4	2.6	17	1	5.9	9	0	0
13. Águeda	34	0	0	17	3	17.6	19	3	15.8
14. Ílhavo	72	3	4.2	37	2	5.4	35	0	0
15. Vagos	29	3	10.3	19	3	15.8	12	2	16.7
16. Oliveira do Bairro	26	1	3.8	22	1	4.5	15	1	6.7
17. Anadia	2	0	0	5	0	0	3	0	0
18. Mealhada	0	-	-	17	0	0	14	0	0
19. Castelo de Paiva	0	-	-	0	-	-	0	-	-

### 3.1. Cats

The sampled feline population exhibited an overall seroprevalence of 8.9%. European Shorthair was the most represented breed (91.5%) followed by Persian (3.5%), Siamese (3.0%) and Scottish Straight (0.9%). No statistically significant differences were observed between groups regarding breed, age, sex, fur length, external deworming, lifestyle or FIV/FeLV status. However, higher seroprevalence was observed in cats aged 11–15 years (15.0%) and 5–10 years (12.9%), as well as in medium-haired (12.8%) and outdoor cats (12.6%). Conversely, unvaccinated cats and those without internal deworming were significantly more susceptible to *D. immitis* infection (Table 2). Vaccinated cats had an odds ratio of being infected of 0.17 (*p* < 0.01) when compared to unvaccinated ones, and internal dewormed ones had an odds ratio of 0.39 (*p* < 0.05) when compared to non-dewormed ones. Regarding lifestyle, our results show that outdoor cats have an odds ratio of 2.72 (*p* < 0.05) of being immunoreactive to *D. immitis*, when compared with strictly indoor or mixed-lifestyle ones.

Considering the 326 sampled cats with a known FIV/FeLV status, we found three cats that were positive for both FIV and FeLV, and only one was seropositive for *D. immitis*, not showing any clinical signs (Table 3). Of the 262 cats negative for both FIV and FeLV, 23 were seropositive and only 10.9% of those had clinical signs. A total of 9.4% (5/53) of the FIV+/FeLV Neg cats were seropositive and only one exhibited clinical signs. We also sampled eight FIV Neg/FeLV+ cats but none of those was seropositive.

### 3.2. Dogs

The overall prevalence in the evaluated canine population (n = 430) was 4.7%. Samples were collected from 36 breeds of dogs. Most of the sampled dogs were mongrels (57.9%), followed by Labrador Retriever (10.5%), Dobermann Pinscher (5.8%), Yorkshire Terrier (4.2%) and German Shepherd (2.3%). No statistically significant differences were noted between breeds. Regarding age, no positive cases were detected in dogs younger than one year or older than 15 years. The highest prevalence (7.0%) occurred in dogs aged 5–10 years, though this was not statistically significant compared to other age groups. Females comprised 50.2% of the population, with no significant differences between sexes, despite a slightly higher prevalence in males. Although prevalence was higher in short- and medium-haired dogs, as well as in non-dewormed dogs; these differences were not statistically significant.

In the evaluated canine population, 64.4% were up to date on vaccination, exhibiting a significantly lower prevalence, with an infection risk of 0.22 (*p* < 0.01), when compared to unvaccinated dogs. Dogs living outdoors had a significantly higher prevalence compared to those with an indoor or mixed lifestyle (Table 4).

### 3.3. Human

Serum samples were collected from individuals residing in the Aveiro district, with an overall seroprevalence of 3.0%. Among the samples, 216 (54.3%) were from females and 182 (45.7%) from males, with ages ranging from 12 to 95 years. The age and gender distribution was representative of the population in Central Portugal, according to the 2021 census data [47], albeit with a slightly overrepresentation of older individuals. Table 5 presents the distribution of samples by gender, age and municipality of residence. Although none of the differences were statistically significant (*p* > 0.05), seroprevalence was slightly higher in males aged 41–65 years. The municipalities with the highest seroprevalence were Vagos (10.3%), Sever do Vouga (7.1%) and Ílhavo (4.2%).

### 3.4. Risk Maps

**Figure 2:**
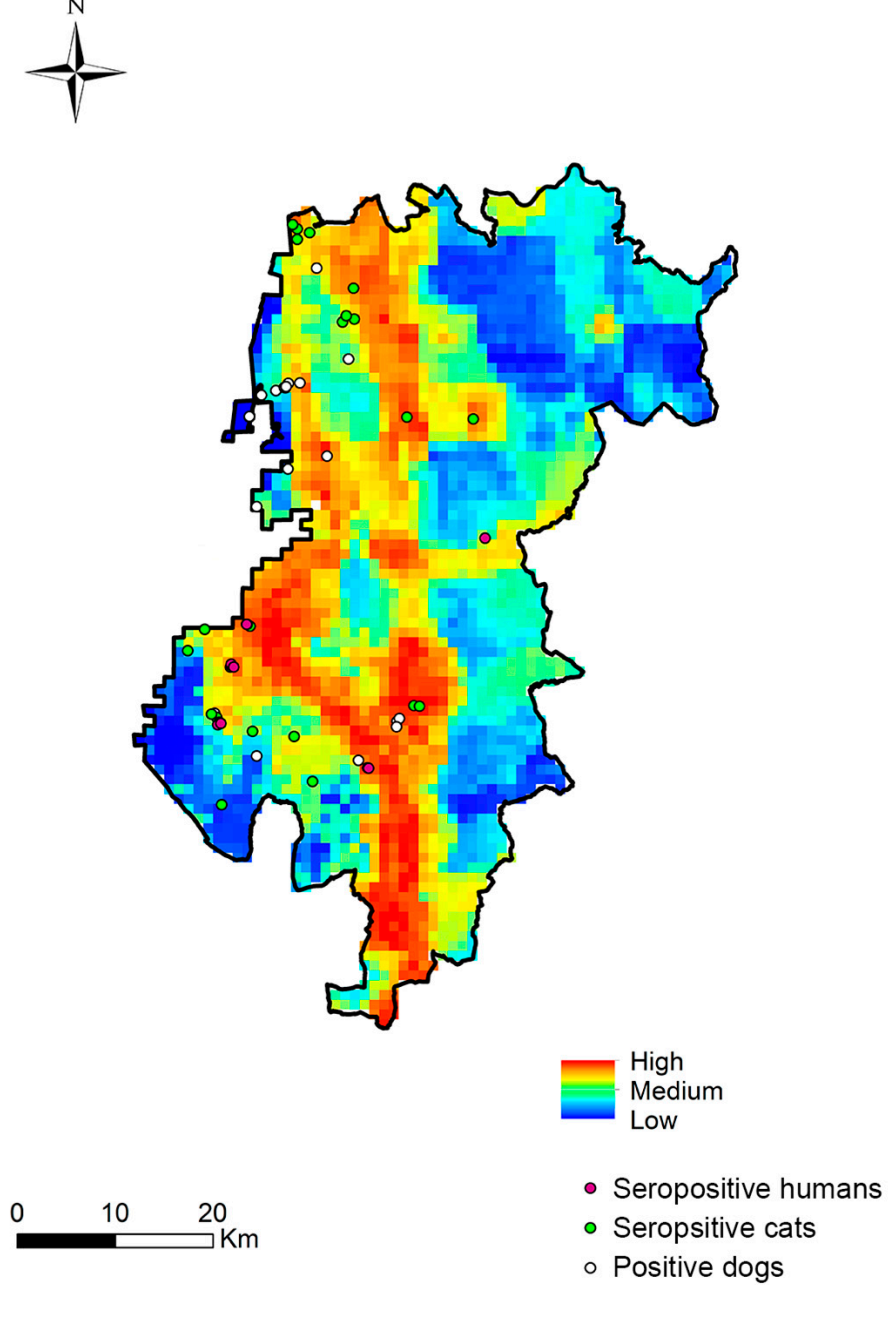
*Dirofilaria immitis* infection risk map with geolocalization of the seropositive humans, cats and infected dogs in Aveiro.

Figure 2 presents a color-coded map depicting the potential risk of *Dirofilaria* spp. transmission in the Aveiro district. The highest infection risk is observed along roads connecting to Porto and Coimbra, in coastal regions with cultivated land, in areas with high concentrations of humans and domestic animals, and at lower altitudes. For the study area, we defined three risk categories—high, medium and low—where 53% of the territory falls within a high-risk zone (red, orange and yellow), 23% in a medium-risk zone (green and light blue) and 24% in a low-risk zone (blue and dark blue). To validate the transmission risk map and assess its predictive power, georeferenced data on *D. immitis*-seropositive humans and cats, and infected dogs were overlaid onto the map. Among the positive samples, 75% of humans, 43.5% of cats and 35% of dogs were in high-risk zones. In medium-risk areas, 25% of humans, 43.5% of cats and 40% of dogs tested positive. Conversely, in low-risk zones, no positive cases were detected in humans, while 13% of cats and 25% of dogs were positive.

## 4. Discussion

Dirofilariosis is an emerging global disease, despite the increased awareness in recent years. While significant research has been conducted on the topic, factors such as global warming, increased international travel with pets and the expansion of invasive mosquito species continue to drive its spread. In dogs, the prolonged asymptomatic phase complicates owner compliance with chemoprophylaxis, often delaying intervention until it is too late. This contributes to the growing reservoir of *D. immitis*, which, in the presence of competent vectors, remains a key factor in disease transmission [48,49].

*Dirofilaria immitis* was first reported in Portugal in 1996 and re-emerged about a decade later in otters from Alentejo [50] and red foxes in Dunas de Mira [51], the latter notably close to the Aveiro district. Since then, no further studies on wild life in Aveiro or its surroundings have been conducted until a 2022 study in Peneda-Gerês National Park, in northern Portugal, confirmed the presence of *D. immtis* in wolves and red foxes [52]. Given the challenges of controlling wild animals and its associated diseases, along with the life cycle of *D. immitis*, wild reservoirs may play a crucial role in disease transmission and should be a focus of future research.

The Aveiro district has a temperate weather with dry or mild summers [42]. Like the rest of the world, it has been affected by climate change over the past decades, with recorded increases of 1 °C in both median and maximum temperatures [43], and projections indicating a further rise of 1–2 °C in the next 30 years [53]. In addition to favorable temperature conditions, Aveiro experiences high humidity levels due to frequent rainfall, its coastal location and the presence of Ria de Aveiro—a 75 km^2^ lagoon system with coastal ridges, marshland and lagoons—providing optimal breeding conditions for mosquitoes [54], the vectors of *D. immitis*. In Portugal, both native mosquito species contribute to disease transmission [55] and invasive species, such as *Aedes albopictus*, have been detected in the Algarve [56] and in Penafiel [24], a municipality in the Porto district, just north of Aveiro.

There is no updated information on native mosquito species in Aveiro’s district. A 2008 report indicated that the predominant mosquito species in the Aveiro/Coimbra region were *Anopheles maculipennis* and *Culex pipiens* [57]. Later, in 2014, the most common mosquito species found in the maritime port of Aveiro were *Culiseta longiareolata*, *Ochlerotatus caspius* and *Culex pipiens* [58]. Among these, only *Culex pipiens* is a known vector of *Dirofilaria immitis* [1].

Effective dirofilariosis control strategies must therefore target not only the parasite in mammalian hosts but also the mosquito species, some of which also transmit other important diseases, including Dengue [56], West Nile virus, Chikungunya, and Leishmaniosis [54].

Canine dirofilariosis in Portugal has fluctuated since its initial identification. Previously endemic regions, such as Madeira [34] and Setúbal [59], have exhibited signs of transmission attenuation, with stable or even declining prevalence in recent years [28]. However, the increasing prevalence in certain areas, including Aveiro [28] and Viana do Castelo [60], as well as the emergence of the first positive cases in regions previously considered free of dirofilariosis, such as Guarda, Castelo Branco and Leiria [28], highlight the concerns about uncontrolled disease spread. Although the overall canine prevalence in Aveiro found in our study (4.7%) was lower than previously reported (15.0% [28]), certain municipalities, such as Águeda (15.8%) and Vagos (16.7%), exhibited even higher prevalences rates. These areas should be prioritized for urgent control measures. This increase in prevalence may be associated with a lack of awareness of the disease among veterinarians and pet owners. Although resistance to macrocyclic lactones has been previously reported in the USA [61] and in a case imported from the USA to Italy [62], studies carried out on European samples do not support this hypothesis [63]. In Madeira and Setúbal, increased awareness among clinicians and the implementation of control measures has effectively mitigated disease spread [28,34,59], with no evidence of pharmacological resistance reported in the country. Conversely, dirofilariosis endemicity in the district of Aveiro is a relatively recent concern, and our study should serve as a tool to raise awareness regarding the presence and dissemination of this disease in the region.

The first reports of feline dirofilariosis in Portugal date back to a decade ago, when the *D. immitis* antigen was detected in 4.8% of sampled cats in the Algarve, in the south [38], and an overall seroprevalence of 15% was found in cats from the north and center [37]. Interestingly, in the latter study, Aveiro had the highest seroprevalence among the analyzed districts (18.7%). In 2020, a study in Madeira Island tested 141 cats for *D. immitis* antigen, revealing a prevalence of 3.5% [39]. More recently, a Mediterranean study reported an overall seroprevalence of 1.7% in Portugal, though it did not specify the regional distribution [64]. To the best of our knowledge, no further data on feline dirofilariosis in Portugal have been published.

Cats can exhibit a wide range of clinical signs, which can influence the diagnosis and treatment of dirofilariosis. If they tolerate the early stages of infection, they may remain asymptomatic or develop chronic respiratory signs (dyspnea, cough, tachypnea or sneezing), resembling primary bronchial disease [5,65]. These signs result from pulmonary endothelial and parenchymal damage caused by worm migration [2]. Due to this overlap, feline dirofilariosis is often misdiagnosed as asthma, leading to inadequate treatment. Heartworm Associated Respiratory Disease (HARD) syndrome has been described as a severe pulmonary inflammatory reaction triggered by the acute death of immature *Dirofilaria* worms upon arrival in the pulmonary circulation [6]. Similarly, the death of the mature worms can lead to thromboembolism, infarction and hemorrhage [2]. Consequently, in some cases, the first and only sign of dirofilariosis in cats is sudden death, with diagnosis occurring incidentally during necropsy [66,67]. The limited routine performance of necropsies may contribute significantly to the underreporting of the disease. In this study, the higher seroprevalence observed in cats compared to dogs suggests that feline dirofilariosis may be largely undiagnosed. This is likely due to the lower adherence to chemoprophylaxis in cats compared to dogs, highlighting the need for increased awareness and preventive strategies.

We observed a significantly lower seroprevalence in vaccinated and internally dewormed cats compared to unvaccinated or non-dewormed ones. This may be partially explained by the effect of milbemycin oxime, a commonly used dewormer for gastrointestinal parasites in cats, which—although not administered monthly as recommended for *D. immitis* prevention—is typically given every three months. Additionally, owners who adhere to vaccination protocols are more likely to follow regular deworming schedules, contributing to lower infection rates. Similar findings were observed in dogs, where prevalence was significantly lower in vaccinated individuals. While this effect may not be directly attributed to vaccination, it likely reflects a broader pattern of responsible pet ownership. Owners who consistently bring their dogs for vaccinations tend to maintain routine veterinary visits and adhere to other preventive healthcare measures. Although regular chemoprophylaxis was an exclusion criterion in this study, dogs receiving routine veterinary care may have undergone occasional deworming, which, even at a lower frequency, could have contributed to reduced infection rates.

Although we did not observe the statistically significant results, there was an increasing seroprevalence with feline age, reaching 15.0% in cats aged 11 to 15 years. This finding aligns with previous reports from Portugal [37] and Gran Canaria [68]. In Madrid [69] and Barcelona [70], similar results were found, despite a slight decrease in seroprevalence among cats aged 9 to 12 years. Regarding the canine population, we found an increasing prevalence until the 5 to 10 years age group, followed by a slight decline thereafter, consistent with earlier reports on other Csb climates [27,28]. This subsequent decrease may be attributed by reduced outdoor activity among older animals, leading to decreased exposure to *D. immitis* vectors.

We found no significant difference between male and female cats, which is consistent with the findings from Gran Canaria [68] and Madrid [69]. This contrasts with previous reports from Portugal [37] and Barcelona [70], where an increased seroprevalence in male cats was observed, potentially due to their more curious and exploratory behavior, which leads them to spend more time outdoors. Similarly, in dogs we did not detect any differences, aligning with earlier reports [27,28].

Although not statistically significant, our results indicate that outdoor cats exhibit a higher seroprevalence (12.6%) compared to those with a mixed lifestyle (9.0%) or strictly indoor cats (5.0%). In our canine population, the differences were statistically significant: strictly outdoor dogs had a higher prevalence (9.9%) than those with a mixed lifestyle (1.9%) and strictly indoor dogs (3.4%). These findings are consistent with the previous data on cats [37,69,70] and dogs [27,28], and may be explained by the increased potential for contact with vectors outdoors. However, it is becoming increasingly common to find these vectors indoors, as evidenced by the results from strictly indoor cats and dogs. Furthermore, animals kept indoors typically receive better care and regular veterinary visits, which may contribute to a reduced risk of infection.

Similar to previous reports [64], we found no statistically significant difference in seroprevalence based on FIV or FeLV status. However, FIV-positive cats exhibited a slightly higher *D. immitis* seroprevalence (10.7%) compared to FIV-negative cats (8.5%). The difference in seroprevalence among FeLV-positive cats was less pronounced (9.1% vs. 8.9% in FeLV-negative cats), but the FeLV-positive population was only one-fifth the size of the FIV-positive group, which may have influenced the results. In contrast, Montoya-Alonso et al. (2022) [44] reported a significant higher seroprevalence in FeLV-positive cats. Given that microfilaremia in cats is rare and transient, it is noteworthy that a case of a FeLV-positive microfilaremic cat has been documented [71], as well as a FeLV-positive cat with an unusual high adult burden—20 worms located near the tricuspid valve [72]. Interestingly, when examining the presence of clinical signs in the feline population with a known FIV/FeLV status, most *D. immitis* seropositive cats showed no clinical signs. Among seropositive and FIV/FeLV-negative cats, 10.9% exhibited clinical signs, compared to 3.4% in the FIV-positive/FeLV-negative group. Although the FeLV-positive population was small (n = 11), the sole *D. immitis* seropositive cat in this group also displayed no clinical signs. Further research focusing on FIV- and FeLV-positive populations would be valuable to deepen our understanding of the potential impact of these retroviruses on the feline immune system and their association with the clinical presentation of dirofilariosis.

Our study identified an overall feline seroprevalence of 8.9%. The municipalities with the highest prevalence were predominantly coastal (Espinho—26.7%) or located along the Ria de Aveiro (Estarreja—16.7%; Vagos—15.8%; Ovar—14.8%). These findings align with the human seroprevalence rates in Vagos (10.3%), Ílhavo (4.2%) and Aveiro (2.6%), as well as canine prevalence rates of 16.7% in Vagos and 5.9% in Ovar and Murtosa, all of which are influenced by the Ria de Aveiro and the Atlantic Ocean. Our results suggest that the humid environment created by these water bodies and the prevailing moist winds may play a significant role in the persistence and transmission of the disease, likely by facilitating optimal conditions for vector reproduction. These findings are consistent with previous reports on humans [40], cats [37] and dogs [28], which indicate that the Aveiro district exhibits higher prevalence rates compared to other regions.

However, we also observed high feline seroprevalence in inland municipalities, such as Águeda (17.6%), Sever do Vouga (14.3%) or Oliveira de Azeméis (7.4%). These findings were accompanied by human seroprevalence rates of 7.1% in Sever do Vouga and 3.8% in Oliveira do Bairro, as well as canine prevalence rates of 15.8% in Águeda, 6.7% in Oliveira do Bairro and 6.5% in Santa Maria da Feira. This pattern may be attributed to the ease of movement between municipalities [73], as many individuals work in coastal areas but reside inland [74], often bringing their pets. Additionally, stagnant water sources, such as storage tanks and pools, commonly found in these municipalities, particularly in tourist areas and second homes owned by emigrants, may contribute to vector proliferation. These water sources, along with urban heat islands and localized microclimates, can accelerate the development of *D. immitis* larvae in mosquitos, even during colder months. The high prevalence in dogs may also play a key role in disease transmission to other species, as they serve as primary reservoirs. Moreover, although positive cases were detected in high-risk zones, 13% of seropositive cats and 25% of infected dogs were found in low-risk areas.

This underscores the urgent need for routine chemoprophylaxis across all municipalities in the Aveiro district. Recruiting animals from certain municipalities proved challenging due to factors such as the lower representation of veterinary activity or reduced compliance, leading to underrepresentation, particularly in feline samples.

*Dirofilaria immitis* infection in humans has been reported worldwide [11,14,75,76], including in Portugal, where a pulmonary nodule was confirmed in a 38-year-old male from Leiria [13], a region geographically close to Aveiro. Our detection of specific immunity confirms the exposure of the human population of Aveiro to infection by *D. immitis*. While a previous study reported a higher seroprevalence [40], the sample was very limited (n = 8), making our results more representative of the Aveiro district. Nevertheless, our human samples were collected from a single hospital center, which, although serving the entire district, primarily receives patients from nearby cities. This may have introduced bias, as some municipalities—particularly those in the northern part of the district, where alternative hospitals are available—were underrepresented. No statistically significant differences were observed between age groups or genders. However, a slightly higher seroprevalence was noted in males aged 41 to 65 years, while individuals over 65 years showed lower prevalence rates. This trend may be attributed to occupational exposure, as individuals aged 40 to 60 years are typically in their peak working years, whereas those over 65 years are generally retired and spend more time indoors, reducing their risk of exposure to infected vectors.

## 5. Conclusions

This study identifies the emergence of a new endemic zone in the Aveiro district, providing evidence of *D. immitis* circulation not only in animal species but also in humans. We observed higher prevalence rates and high-risk areas near the coast and along the branches of the Ria de Aveiro, as well as in more inland municipalities, highlighting the spread of the disease throughout the entire district.

Raising awareness of dirofilariosis across all affected species is urgently needed. While canine cases are presumably more studied, the rising prevalence indicates that prophylactic, vector control and treatment measures remain insufficient. Feline cases, though less frequently diagnosed, are confirmed by our study. Despite often being asymptomatic or presenting with nonspecific clinical signs, feline infections can have severe consequences, including sudden death. Furthermore, our findings confirm the circulation of *D. immitis* in humans, reinforcing that this disease is no longer solely a veterinary concern in the Aveiro district, but also a significant public health issue.

## Figures and Tables

**Table 2:** Statistical analysis of the variables studied in cats (age, sex, fur length, vaccination, internal and external deworming, lifestyle and FIV and FeLV status). Abbreviations: n = number of sampled cats; % = percentage of sampled/positive cats; * = significant differences between groups.

		Test Result				
		Total		Positive		*p*-Value
		n	%	n	%	
Age ^†^	<1	28	6.6	1	3.6	0.100
	1–4	221	51.9	14	6.3	
	5–10	124	29.1	16	12.9	
	11–15	40	9.4	6	15.0	
	>15	10	2.3	0	0	
Sex	Female	222	52.1	20	9.0	1.0
	Male	204	47.9	18	8.8	
Fur Length	Short	362	85.0	32	8.8	0.497
	Medium	39	9.1	5	12.8	
	Long	25	5.9	1	4.0	
Vaccination	No	291	68.3	35	12.0	0.001 *
	Yes	135	31.7	3	2.2	
Internal Deworming	No	261	61.3	30	11.5	0.030 *
	Yes	165	38.7	8	4.8	
External Deworming	No	326	76.5	30	9.2	0.866
	Yes	100	23.5	8	8.0	
Lifestyle	Indoor	119	27.9	6	5.0	0.123
	Mixed	188	44.1	17	9.0	
	Outdoor	119	27.9	15	12.6	
FIV Status ^†^	No	271	63.6	23	8.5	0.606
	Yes	56	13.1	6	10.7	
FeLV Status ^†^	No	315	73.9	28	8.9	1.0
	Yes	11	2.6	1	9.1	

^†^ Age was unknown for 3 samples, FIV status for 99 samples and FeLV status for 100 samples.

**Table 3:** Relation between FIV and/or FeLV status, the presence of clinical signs and the seroprevalence in the sampled feline population. The FIV status of 99 samples and FeLV status of 100 are unknown. Abbreviations: n = number of sampled cats; % = percentage of sampled/positive cats.

		Results			
		Total		Positive	
		n	%	n	%
FIV Neg/FeLV Neg		262	61.5	23	9.4
	Clinical Signs	46	17.6	5	10.9
FIV+/FeLV+		3	0.7	1	33.3
	Clinical Signs	1	33.3	0	0.0
FIV+/FeLV Neg		53	12.4	5	9.4
	Clinical Signs	29	54.7	1	3.4
FIV Neg/FeLV+		8	1.9	0	0.0
	Clinical Signs	6	75.0	0	0.0

**Table 4:** Statistical analysis of the variables studied in dogs (age, sex, fur length, vaccination, internal and external deworming and lifestyle). Abbreviations: n = number of sampled dogs; % = percentage of sampled/positive dogs; * = significant differences between groups analyzed.

		Test Result				
		Total		Positive		*p*-Value
		n	%	n	%	
Age	<1	6	1.4	0	0	0.325
	1–4	132	30.7	3	2.3	
	5–10	201	46.7	14	7.0	
	11–15	85	19.8	3	3.5	
	>15	6	1.4	0	0	
Sex	Female	216	50.2	8	3.7	0.479
	Male	214	49.8	12	5.6	
Fur Length	Short	251	58.4	14	5.6	0.460
	Medium	144	33.5	6	4.2	
	Long	35	8.1	0	0	
Vaccination	No	153	35.6	14	9.1	0.002 *
	Yes	277	64.4	6	2.2	
Internal Deworming ^†^	No	256	59.5	16	6.2	0.106
	Yes	169	39.3	4	2.4	
External Deworming	No	237	55.1	15	6.3	0.109
	Yes	193	44.9	5	2.6	
Lifestyle	Indoor	29	6.7	1	3.4	0.002 *
	Mixed	259	60.2	5	1.9	
	Outdoor	142	33.0	14	9.9	

^†^ Internal deworming was unknown for 5 samples.

**Table 5:** Human seroprevalence of *Dirofilaria immitis* and its distribution by gender, age and municipality of residence. The municipalities with no samples are not shown. Abbreviations: n = number of sampled humans; + = number of positive specimens; % = percentage of positive specimens.

		Male			Female			Total		
		n	+	%	n	+	%	n	+	%
Overall		182	6	3.3	216	6	2.8	398	12	3.0
Age	<18	3	0	0	3	0	0	6	0	0
	18–40	14	0	0	63	2	3.2	77	2	2.6
	41–65	80	4	5.0	87	3	3.4	167	7	4.2
	>65	85	2	2.3	63	1	1.6	148	3	2.0
**Municipalities**										
Santa Maria da Feira		1	0	0	1	0	0	2	0	0
Ovar		0	0	0	1	0	0	1	0	0
Oliveira de Azeméis		1	0	0	0	0	0	1	0	0
Murtosa		2	0	0	6	0	0	8	0	0
Estarreja		6	0	0	9	0	0	15	0	0
Albergaria-a-Velha		17	0	0	23	0	0	40	0	0
Sever do Vouga		6	0	0	8	1	12.5	14	1	7.1
Aveiro		65	3	4.6	89	1	1.1	154	4	2.6
Águeda		16	0	0	18	0	0	34	0	0
Ílhavo		43	2	4.6	29	1	3.4	72	3	4.2
Vagos		11	1	9.1	18	2	11.1	29	3	10.3
Oliveira do Bairro		14	0	0	12	1	8.3	26	1	3.8
Anadia		0	0	0	2	0	0	2	0	0

## Data Availability

The data presented in this study are available on request from the corresponding authors.
